# Piezo-ionic Materials and Structures for Complex Shear Field Monitoring

**DOI:** 10.1021/acsami.5c10061

**Published:** 2025-07-07

**Authors:** Dong-hee Kang, Jinyoung Kim, Sergio Gonzalez Munoz, Jisoo Jeon, Sehyun Park, Vladimir V. Tsukruk

**Affiliations:** School of Materials Science and Engineering, 1372 Georgia Institute of Technology, Atlanta, Georgia 30332, United States

**Keywords:** shear force sensors, soft electronics, wearable electronics, piezo-ionic force sensing matrix, shoe insole sensors

## Abstract

Capturing shearing stresses is crucial for accurately monitoring multidirectional mechanical deformation, enabling advanced motion analysis and enhancing functionality in wearable and robotic applications. Here, we present self-powered porous piezo-ionic shear-sensing materials that simultaneously resolve normal and tangential stresses with high sensitivity, a broad dynamic range, and high linearity. A composite of thermoplastic urethane and ionic liquids, reinforced with silica nanoparticles, was exploited to establish and sustain a porous framework. This configuration amplifies piezo-ionic responsiveness and broadens the response range. The materials design uses a stacked two-layered architecture with one reversed trapezoidal layer interlocked with a second macrodome layer. Such an arrangement of elastomeric structural elements converts macroscopic shearing deformation into localized directional compression while providing a four-electrode multidirectional signal readout. This hierarchical macro-to-nano structure achieves a sensitivity of 0.23 mV kPa^–1^ and offers reliable and linear performance over a broad pressure range (up to 634 kPa), a critical advantage for continuous monitoring applications, compared to conventional piezo-ionic materials which often exhibit saturation at high loads. Integrated into a thin single-stack film, these sensors can map complex gait patterns, including walking, pivoting, and tiptoeing, with low power consumption and scalable fabrication, establishing a universal platform for high-fidelity shear monitoring in wearable robotics and industrial friction sensing.

## Graphical abstract

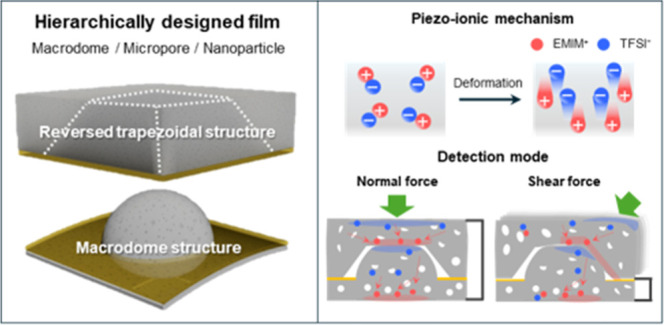

## Introduction

Elastomeric shear-force sensors are essential for sensing tangential loads that govern performance or safety, such as detecting subtle foot slip in gait analysis,^1–3^ enabling dexterous manipulation in robotic grippers,^4–6^ providing haptic feedback in virtual and augmented reality,^7,8^ and monitoring frictional forces in automated assembly lines.^9^ Unlike traditional pressure sensors, shear-detecting devices must resolve both the magnitude and direction of lateral stresses, decouple them from normal loads, and maintain high sensitivity over a wide dynamic loading range. In addition, a suitable structural materials design with low-power consumption and mechanical robustness is a crucial point to develop a sensitive and wide-range load shear force monitoring for wearable and soft robotics applications.

Still, today’s shear sensors grapple with significant hurdles. Many designs saturate at relatively low tangential forces,^10,11^ severely limiting their usable detection window. Decoupling shear from normal pressure typically relies on stacked multilayer architectures^12^ or complex signal-processing algorithms,^13,14^ increasing materials system complexity. Achieving high sensitivity across a broad dynamic range remains elusive, as well. Many sensors that detect minute shear shifts lose linearity when subjected to higher loads and vice versa. Moreover, cyclic loading introduces hysteresis and material fatigue, compromising the repeatability over time. Finally, fabricating multiaxis shear arrays often involves intricate microstructuring or clean-room processes, driving up production costs and restricting scalability.^15,16^

Recent studies have underlined the need for integrated sensors capable of capturing both normal and shear forces with a low power consumption. Various mechanisms—capacitive,^17,18^ resistive,^3,4,19^ triboelectric,^20,21^ magnetic,^22^ and piezo-ionic^23,24^ —have been explored for multiaxial force detection. While capacitive and resistive sensors show potential, they suffer from power supply limitations. Triboelectric sensors can detect only dynamic pressure, which is shown as two sharp peaks with positive and negative values at only loading and unloading points. This unique property makes them unsuitable for continuous pressure monitoring, especially with an increase in the amount of pressure without unloading.

Among these, piezo-ionic sensors offer notable advantages, including ultrahigh sensitivity by virtue of the electrical double-layer effect at the electrode–electrolyte interface and inherently low power consumption, which is especially suitable for wearable applications. Bai et al.^4^ introduced piezo-ionic graded interlocks to extend sensitivity and present a hierarchical microstructure for wide-range, linear pressure sensing. Still, they used the power supply to operate the sensor and showed pressure sensing capability only at normal pressure, which leaves room for future development of shear force sensors. Expanding the sensing range enables accurate capturing and prevention of early saturation and facilitates measurements across diverse applications, from gentle human–machine interactions to industrial pressure monitoring.

Among recent materials developments, porous dielectric elastomer-based approaches by Ham et al.^25^ differentiate directional components of force but only at low loading levels. Wang et al.^12^ fabricated a porous, ionic composite to monitor human movement but confined their detection to lower pressure. Many existing sensor designs concentrate on normal-pressure detection, while tangential force detection often experiences quick saturation at low loads. To enlarge the sensing range, a sensing material matrix with dynamic deformation and durability is needed.

In this study, we propose a novel material design based upon a porous matrix reinforced with silica nanoparticles, which enhances film stiffness and supports porous morphology and broadens the pressure detection range. Using the ionic liquid inside the polymer matrix enhances ionic conductivity and diffusivity.^26,27^ Moreover, adding mechanically dissimilar materials promotes localized stress concentrations that can be alleviated by the hierarchical architecture from the macro-to nanoscale, thereby elevating sensor sensitivity. Overall, a shear force sensor design suggested here with a robust, interlocked configuration of a reversed trapezoidal structure and a macrodome structure facilitates concurrent measurement of normal and shear pressures with enhanced linear sensing performance across a wide operating range that allows monitoring of complex human motion.

## Results and Discussion

### Design and Working Mechanism

A piezo-ionic shear sensor is based on a porous thermoplastic polyurethane (TPU) matrix incorporating an ionic liquid (EMIM-TFSI) and silica nanoparticles (SiNPs) (Figure 1a). The ionic liquid works as a signal-generating material by its inherent properties of dissociation in response to deformation of the composite matrix. A porous matrix with SiNPs can further enhance the response to applied pressure to a larger extent of deformation within the structure itself.

**Figure 1:**
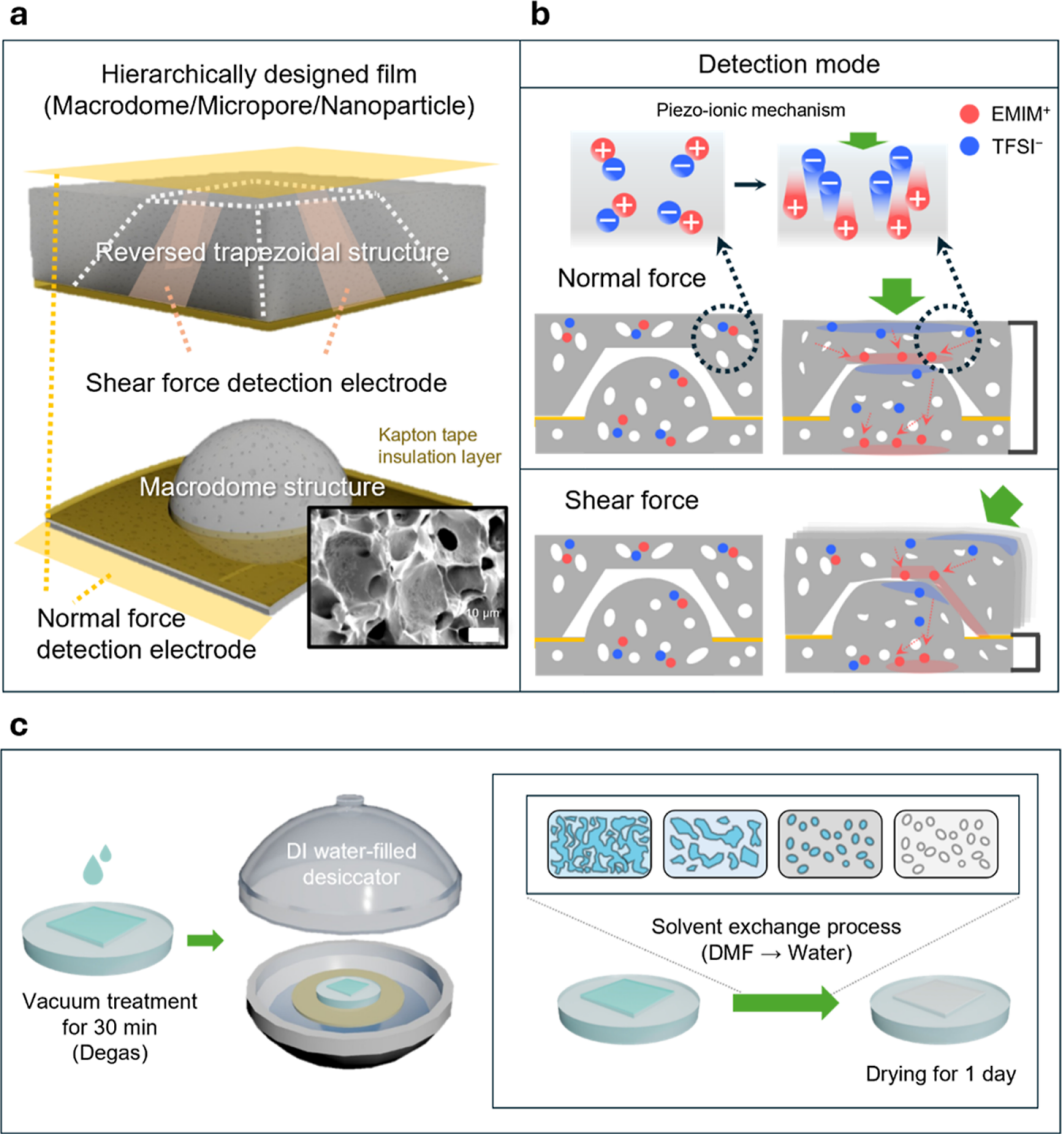
Shear force-sensing materials and systems. (a) Illustration of the structure and materials design for the shear force sensor. Two hierarchically structured porous TPU/ionic liquid/SiNPs films with reversed trapezoidal/macrodome pattern (scale bar = 10 μm). (b) Schematics of piezo-ionic detection mechanisms of different forces. Ionic liquid components with different charges are dissociated in a deformed state. Differently connected electrodes from different sites of structures can selectively respond to the normal force and shear force. (c) Schematics of a fabrication process of an elastomeric porous matrix. After pouring on the PDMS mold, the solution undergoes a solvent exchange process inside a water-filled desiccator.

The top surface features a reversed trapezoidal geometry patterned with four sloped electrodes and a top central electrode for normal force detection. The opposite side comprises a rounded macrodome that serves as the ground electrode. These interlocking layers are spatially separated by a Kapton spacer to ensure an independent contact. Within the porous matrix (Inset of Figure 1a), SiNPs are homogeneously dispersed throughout the TPU and ionic liquid (EMIM–TFSI) matrix.

The sensing mechanism is governed by the piezo-ionic effect that relies on ion-pair dissociation and subsequent potential generation across the electrode–electrolyte interface under mechanical deformation induced volumetric strain (Figure 1b).^28^ The piezo-ionic phenomenon fundamentally relies on the difference in mobility between cations and anions during ion redistribution triggered by external mechanical stimuli such as pressure. When such stimuli are applied, ion convection is initiated within the material, resulting in an asymmetric flow of ions—typically favoring one charge over the other.^29^ This movement leads to a localized charge imbalance, producing a polarization effect similar to the Donnan potential.^30^ Unlike conventional self-powered sensing mechanisms like piezoelectricity or triboelectricity, which generate transient electrical signals in response to dynamic stimuli, piezo-ionic materials can monitor static or gradually changing forces without requiring an external power supply. This enables them to operate continuously under sustained stimuli. The mismatch in mechanical modulus between the silica nanoparticles and the surrounding polymer matrix induces localized stress concentration;^31,32^ thereby, ion dissociation under mechanical stimuli can happen more actively, which leads to enhancing the sensing performance.

In normal force detection mode, the electrode at the top of the reversed trapezoidal structure and the bottom of the macrodome structure are connected. The potential difference between the two vertically aligned electrodes is measured at this connection. For shear force detection, the electrode at the slope of the trapezoidal structure is connected to the bottom electrode of the macrodome structure, which can measure the applied shear forces in different directions.

### Fabrication and Materials Design

The thermoplastic polyurethane material was selected as the primary matrix material due to its excellent flexibility and long-term mechanical stability, which are critical for pressure sensors subjected to continuous dynamic loading. We fabricated a porous matrix with both mechanical robustness and high deformability using a solvent exchange method^16^ in Figure 1c (see the detailed fabrication process in the Methods Section).

Briefly, a mixture containing TPU, the ionic liquid EMIM-TFSI, and SiNPs is cast onto two distinctively patterned (macrodome, reversed trapezoidal structure) polydimethylsiloxane (PDMS) molds. The PDMS mold was fabricated through replica molding using a 3D-printed master structure. After casting the solution onto the PDMS mold, the mold stays inside the desiccator with DI water. The solution inside the PDMS mold undergoes phase separation between solvent (DMF) and nonsolvent (water) components during the solvent exchange processes (inset of Figure 1c).^33^ Exposing composite solutions inside a humid condition can condense water droplets on their surface, resulting in a film soaked with water after solvent evaporation (see the picture in Figure S1).

The pores formed inside the film matrix showed a broad size distribution from 10 to 20 μm. As observed, SiNPs preferentially adhered to the inner surfaces of the pores (Figure 2a). We conducted drying tests to evaluate the effects of different SiNP contents (0, 1, 3, and 5 wt %) on moisture response and structural stability (Figure 2b). For these tests, we soaked each sample in water for 5 s and then monitored weight loss over time to determine evaporation speed. Under these conditions, the sample containing 3 wt % SiNP exhibited the fastest drying time (26 min), significantly outperforming the 0 and 1 wt % samples, which took 60 and 45 min, respectively. The rapid drying capability is crucial as the fabricated porous film must dry easily and avoid prolonged wetting.

**Figure 2:**
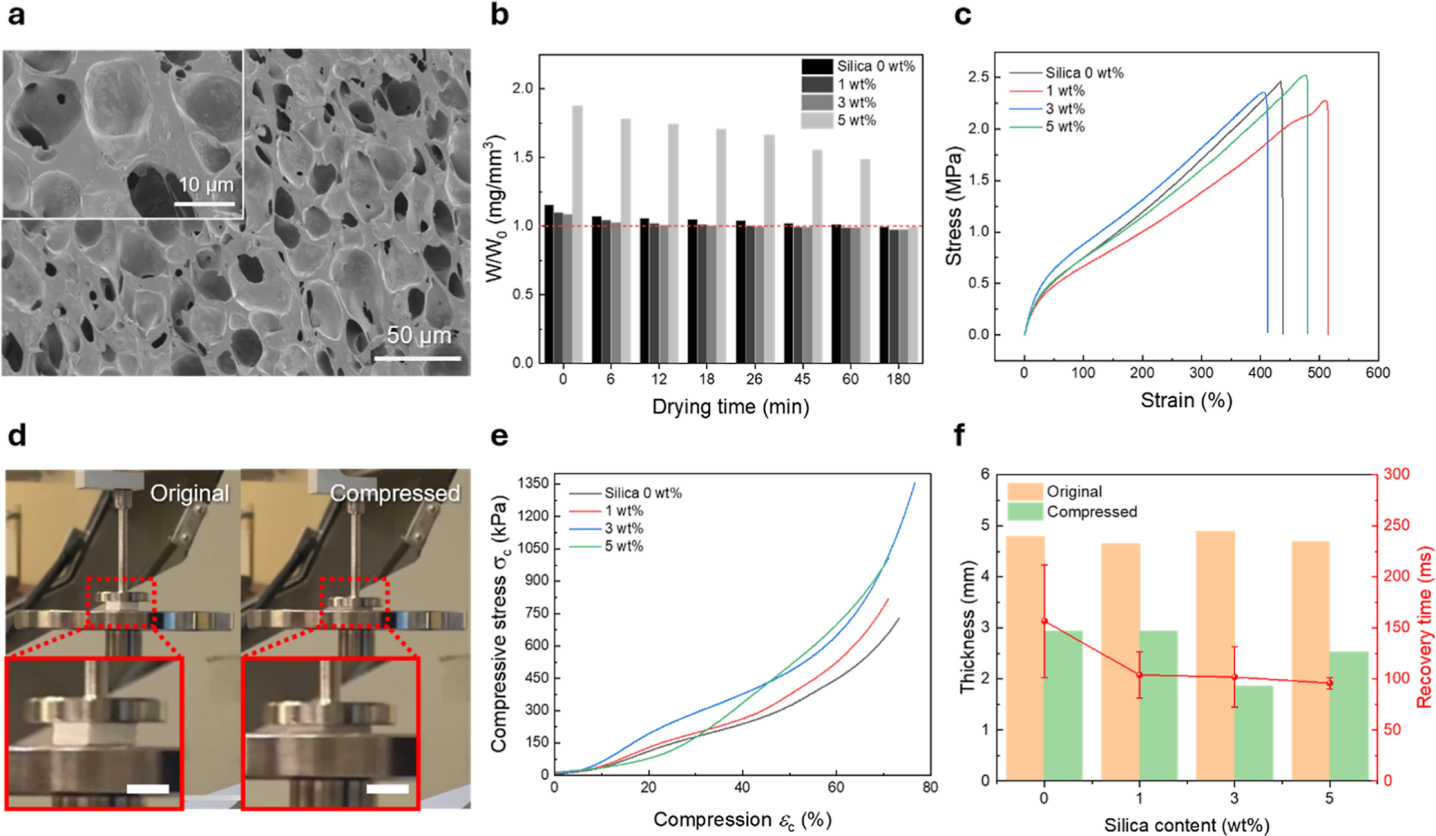
Fabrication process and mechanical properties of composite porous films. (a) SEM image of the composite porous matrix. (b) Drying test of the fabricated porous matrix at different SiNPs NP concentrations and comparison of weight after soaking into water for 5 s. (c) Tensile test results of fabricated porous films. (d) Digital image of the compression test (scale bar = 5 mm). (e) Measurement result of the compression test at different SiNP contents. (f) Thickness change of the fabricated porous film at its full compression and its recovery time to return to its original thickness.

These results suggest that a moderate silica nanoparticle loading not only reinforces the structure but also promotes water evaporation by maintaining the pore architecture. The open pore structure with a robust porous structure enhances water evaporation. However, when the amount of SiNP exceeds a moderate amount, 5 wt %, the film captures water inside the pore structure because of the aggregated SiNPs, which make it harder to evaporate.

Figure 2d is the picture taken from the compression test of the fabricated porous film. The 3 wt % SiNPs film required the highest force to reach full compression (Figure 2e), confirming enhanced mechanical integrity. In addition, recovery after compression is also a crucial property for the shoe insole application. For an accurate and repetitive measurement of pressure stimuli, fast recovery without changing the original dimension is ideal for a fabricated sensor. As we add the SiNPs inside the porous matrix, the recovery time from full compression to its original thickness decreases to half (264 ms at SiNPs 0 wt % to 102 ms at SiNPs 3 wt %), indicating suitability for dynamic mechanical response (Figure 2f).

Tensile mechanical testing showed elastic moduli of 21.08, 19.64, 24.75, and 18.49 kPa for films containing 0, 1, 3, and 5 wt % SiNP, respectively (Figure 2c). The 3 wt % sample demonstrated the highest stiffness, attributed to the optimized particle–polymer interaction and structural reinforcement. The decreasing elastic modulus of the 5 wt % sample is due to the aggregation of SiNPs in the TPU matrix.

## Materials Dynamics under Normal Pressure

The fabricated sensor operates with a piezo-ionic mechanism, which is based on the potential difference created between two different layers due to ion dissociation from mechanical stimuli (Figure 3a). These layers with varying potentials inside the film are named as the electron double layer (EDL).^34^

**Figure 3:**
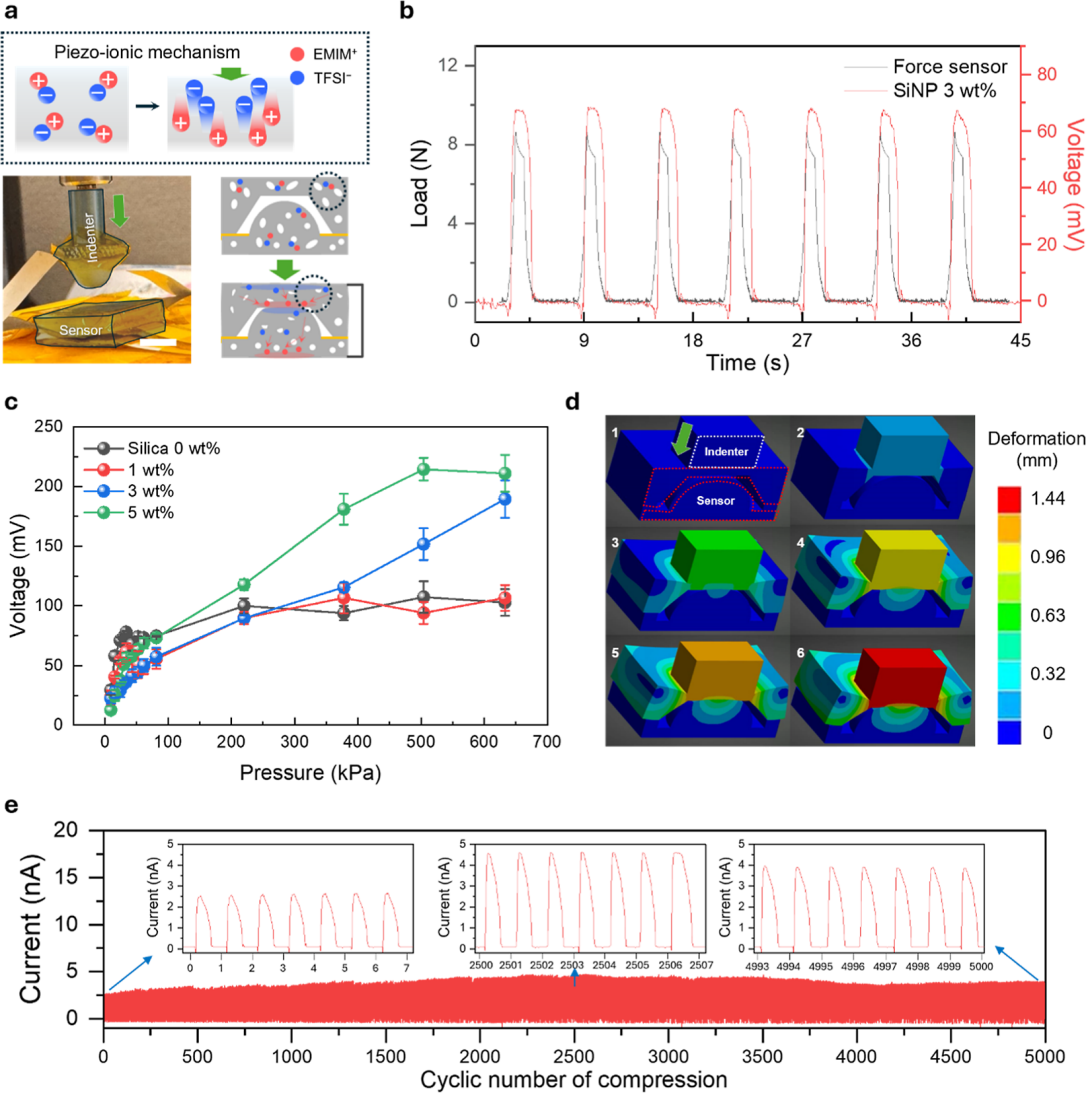
Normal force measurement of the fabricated sensor system. (a) (Top) The schematics showing the piezo-ionic mechanism (lower left); digital picture of the sensor and a normal force measurement tip. Scale bar = 0.5 cm (lower right). The schematic showing ion dissociation upon loading a normal force to the fabricated sensor. (b) Result of the force sensor of the measurement system during a loading–unloading cycle. (c) Voltage output result of the fabricated sensor system with different amounts of SiNPs. (d) FEM simulation models demonstrating deformation when normal pressure is applied to the fabricated sensor system. Deformation was evaluated under a normal force ramping from 0 to 20 N over 1 s: 1–0 s, 0 N; 2–0.105 s, 2.1 N; 3–0.368 s, 7.36 N; 4–0.736 s, 14.72 N; 5–0.842 s, 16.84 N; 6–1 s. 20 N. (e) Result of voltage output at measuring the repetitive response of the fabricated sensor system.

When mechanical pressure is applied, the resultant volumetric changes within the film induce dynamic movement of ions from the ionic liquid. Specifically, cations often move faster than anions, leading to a temporary charge separation and thus a potential difference across the film. This ionic reconfiguration reverses as the pressure stimulus is removed, thus enabling self-powered operation.^29^ To evaluate the response of the fabricated porous piezo-ionic sensor under normal loading conditions, we conducted a series of mechanical and electrical measurements using a customized compression setup (lower left picture of Figure 3a). This setup is composed of a round indenter made from a hard polyurethane-based resin, while the fabricated sensor is firmly fixed on a stage beneath it to enable precise normal force detection.

For system calibration, the force response of a commercial force sensor was measured under identical conditions, serving as a reference to validate the applied pressure profile and relate to a real-time voltage outcome (Figure 3b). The response time of the fabricated sensor is 300 ms in response to an applied force of 8.8 N at a rate of 250 mm/s (Figure S2a,b).

Materials with varying SiNPs content (0, 1, 3, and 5 wt %) were tested under increasing applied forces as noted above (Figure 3c). The detailed plot of the lower-pressure region is shown in Figure S3. The sensor with 3 wt % SiNPs exhibited the most linear and extended voltage response, showing a sensitivity of 0.229 mV/kPa up to 633.5 kPa of applied pressure. In contrast, softer structures with 0, 1, and 5 wt % SiNPs displayed early signal saturation. To convert the applied force to the applied pressure, the contact area of the cap is calculated. The cap distance is measured by applying different forces to a porous planar film (Figure S4a–c). Moreover, the sensor demonstrates consistent and reproducible voltage output across multiple cycles within the same normal pressure range from 2.5 to 100.9 N (Figure S5). The stable cyclic response confirms the film’s resilience under repeated mechanical deformation and its suitability for real-time pressure monitoring. To confirm the fabricated sensor’s ability to decouple normal and shear forces, we evaluated the signals from the shear force connections while applying only normal force. Figure S6 presents the sensor’s response with different electrode connections under a 10 N normal force. Figure S6a schematically illustrates these distinct electrical connections within the sensor. As a normal force is applied to the sensor, characteristic pressure sensing peaks are generated. Crucially, the shear force connection shows no significant response, exhibiting only negligible fluctuations, thus confirming that unintended contact or signal generation from shear electrodes does not occur during normal force measurement (Figure S6b,c).

To gain insight into stress distribution within the sensor architecture, finite element modeling (FEM) was performed to model deformations of fabricated structures during compression (Figure 3d, see the Supporting Information). Under a normal pressure of 20 N, the simulation revealed a progressive contact area expansion and stress redistribution as the film deformed toward full compression. From this result, we can see that the progressive contact area increases as the applied pressure increases, which results in increased volumetric expansion and dissociation of the ionic liquid inside the porous film.

Finally, the fabricated sensor shows a stable and nondegrading signal output throughout the 5000 loading–unloading cycles under 8 N of force (Figure 3e). A slight initial increase was attributed to ionic pathway stabilization. Thus, a 3 wt % SiNPs composition offers an optimal balance between mechanical stiffness and ion mobility within the reinforced porous elastomeric matrix.

### Mechanical Behavior under Shear Force

In practical pressure-sensing conditions, the shear force sensor is typically subjected to an initial preload due to the weight of the wearer, even under static conditions. To simulate these real-world conditions and evaluate shearing behavior with a normal pressure preload, we established a test setup incorporating both vertical and horizontal force components (Figure 4a). The setup consisted of a fixed sensor stage and a horizontally actuated syringe pump equipped with a preload mass. The preload is fixed to the glass covering the top of the fabricated sensor. The applied shear force is aligned precisely with the intended shear force direction. The syringe pump applied shear motion at varying speeds, while static weights (0.98 to 9.8 N), which are attached to a planar indenter, were placed on top of the sensor to introduce controlled preloads. After the indenter fully proceeds to its maximum distance, the pump returns to its original position.

**Figure 4:**
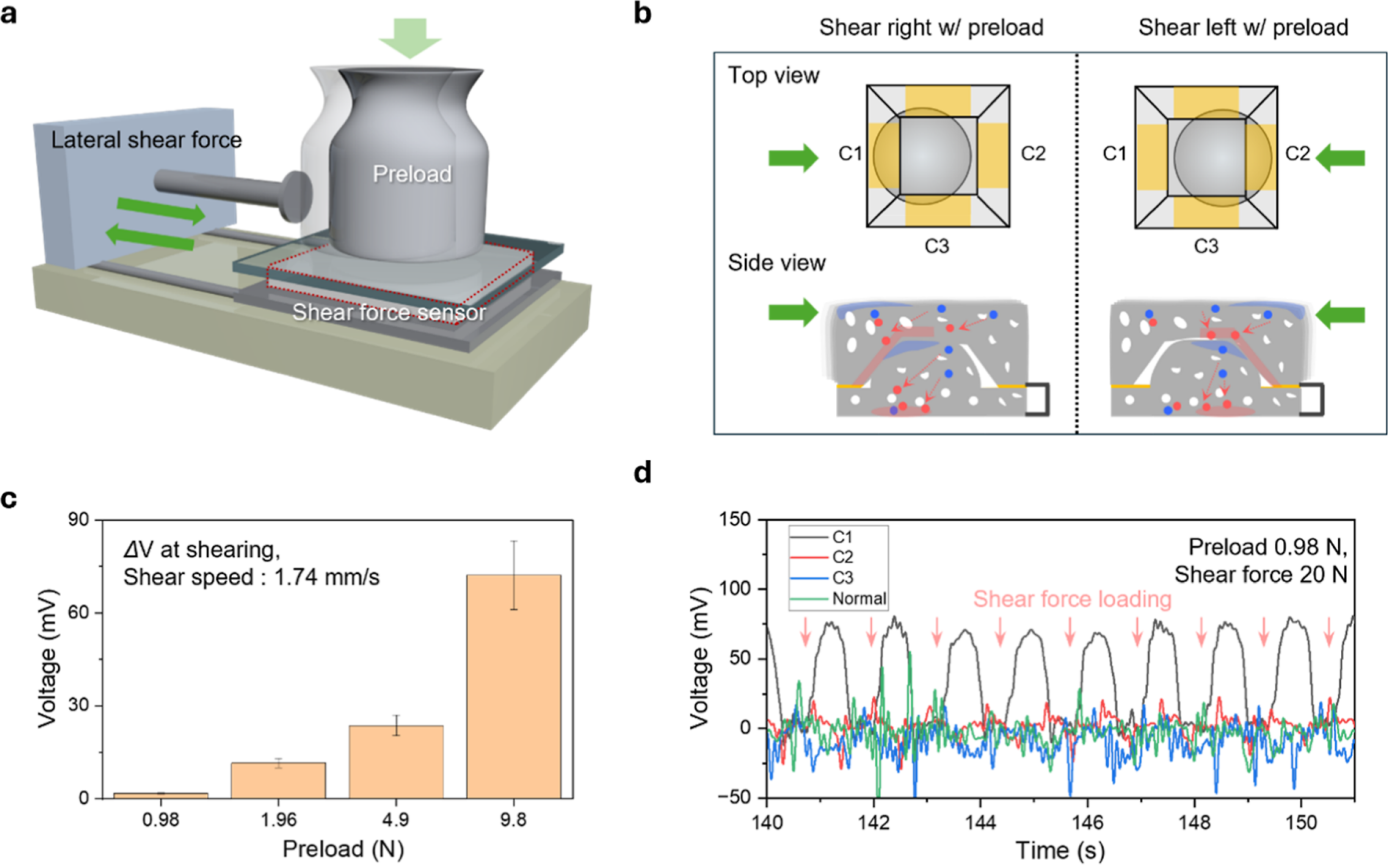
Shear force behavior with preload conditions. (a) Schematics showing the measurement setup of shear force measurement with normal preload. (b) Schematics of a top and cross-sectional view of the fabricated sensor structure while applying normal preload and shear force with preload. The electrodes in different directions of the slope of the reversed trapezoid structure are named C1, C2, and C3. Signals are generated in only the shear direction. (c) Measurement results of applying shear force at different preloads from 0.98 to 9.8 N. (d) Shear force measurement results at preload conditions (0.98 N) with different electrode connections.

The top and cross-sectional schematics of the sensor deformation under these loading conditions are shown in Figure 4b. When the preload is applied, the macrodome and reversed trapezoidal structures establish baseline contact, responding to a static normal pressure. A subsequent horizontal shear force then alters the contact pattern asymmetrically, generating a differential voltage across the patterned electrodes. Directional force sensitivity was monitored by connecting different electrodes on the trapezoidal structure and applying a shear force along a fixed direction. On the top view of the fabricated sensor structure, electrodes for collecting output were denoted as C1 (shear direction), C2 (reverse direction), and C3 (orthogonal side direction).

As shown in Figure 4c, the magnitude of voltage output from electrode C1 increased with greater preload mass (0.98, 1.96, 4.9, and 9.8 N) at fixed shear speed and direction (right), indicating that the interaction between the two patterned films enhances the shear response at higher preload conditions. Figure 4d presents the electrical responses at different electrode connections under applied shear force and preload conditions. The response associated with shear loading in the right direction is highlighted by a red arrow within the plot. Using the experimental configuration depicted in Figure 4a, a rightward 20 N shear force was applied to the sensor, reaching the maximum displacement under preload conditions and subsequently returning to its initial position. Electrodes C1 exhibited positive responses corresponding directly to the rightward shear force, whereas electrode C2 and C3 generated relatively sharp signals predominantly during the unloading phase, specifically at the moment when the preload motion stopped and returned to the original position.

For a quantitative measurement of shear force, we applied varying shear forces (3, 8, 12, and 20 N) to the fabricated sensor. Shear force is expressed in Newtons, aligning with the direct measurement of the applied tangential load and common practice in characterizing discrete force sensor elements.^16,17^ Figure S7 presents the voltage response from the different electrode connections (C1, C2, and C3) and the normal connection, showing a gradual increase in signals with increasing applied shear force. To precisely analyze the decoupling of shear and normal forces, we conducted measurements using distinct connections for normal and shear force detection (Figure S8). Figure S8a schematically illustrates these different connections under the applied shear force condition. Figure S8b plots the comparative response of the shear and normal force connections when a 20 N shear force is applied under a 0.98 N preload. A detailed peak analysis in Figure S8c clearly shows a prominent shear peak, while only minor fluctuations are observed in the normal force connection electrode signal, confirming effective decoupling under the given preload conditions.

### Shear Force Induced with an Angular Stage for Directional Force Detection

To further assess the directional sensitivity and performance under combined normal and shear loads, we implemented an angular loading platform that mimics oblique force application, which frequently occurs during walking, pivoting, or moving on inclined surfaces. The custom-designed angular stage was fabricated via filament-based 3D printing, incorporating inclined surfaces at 10°, 20°, and 30° (Figure 5a). Shear force sensors were fixed onto each angular plane, and a round-tip indenter was used to apply vertical compression.

**Figure 5:**
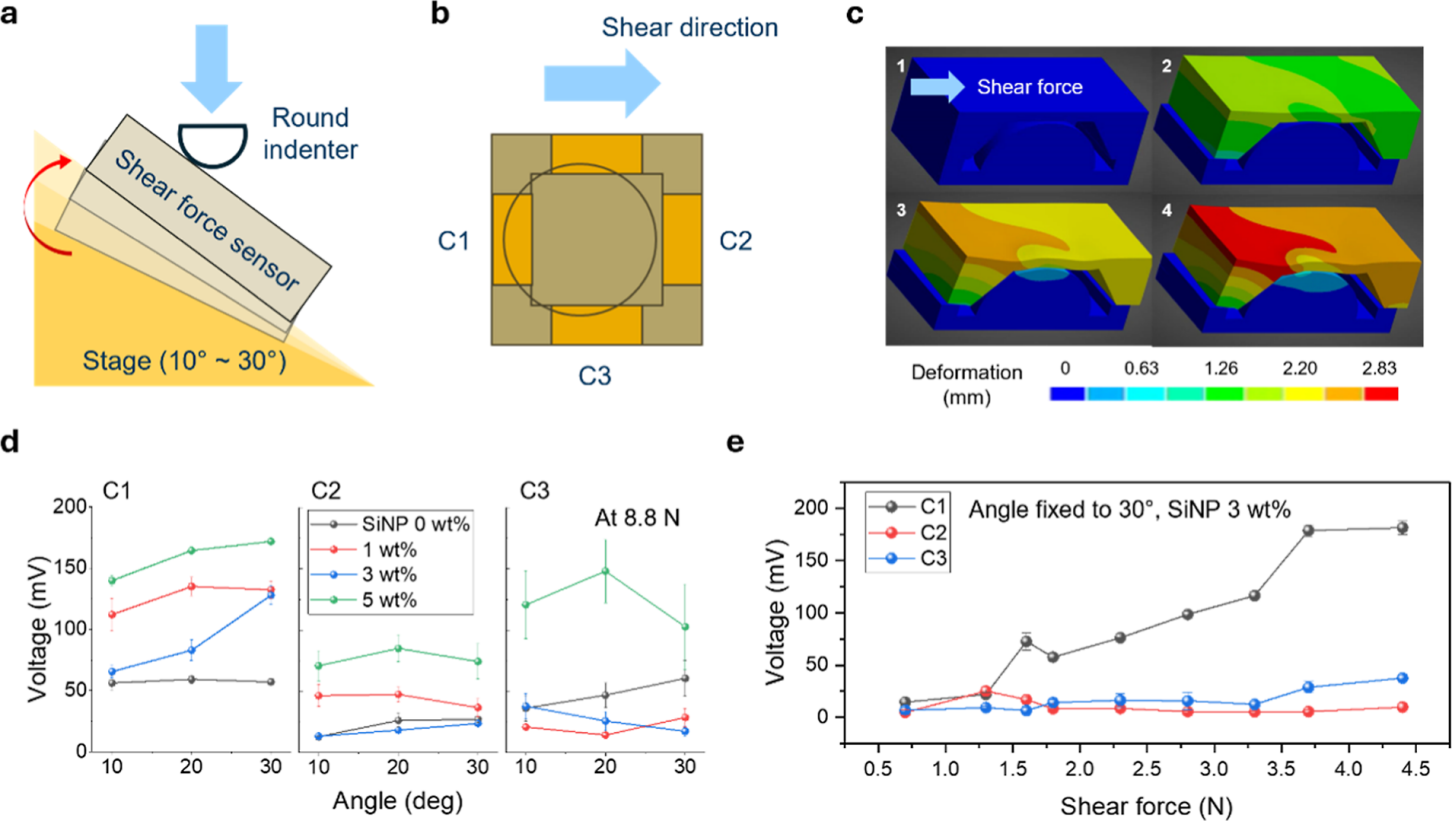
Combined normal and shear force measurement at the angular stage. (a) Schematic illustration of combined normal and shear force measurement at the angular stage. (b) Schematic illustration of the inside of the shear force sensor while experiencing combined normal and shear force at the angular stage. The shear direction (C1) and side direction (C3) show signals while applying shear force. (c) FEA simulation of deformation of the fabricated sensor at the angular stage, having an angle of 30°. Deformation states were evaluated from 0 to 20 N over 1 s: 1–0 s, 0 N; 2–0.263 s, 5.26 N; 3–0.736 s, 14.72 N; 4–1 s, 20 N. (d) Response at different electrode connections (C1, C2, and C3) of fabricated shear force sensors with different amounts of SiNPs content (0, 1, 3, 5 wt %) at increasing angles of angular stage to 10, 20, and 30°; the applied force is fixed to 8.8 N. (e) Directional response of the fabricated shear force sensor at a fixed angle of 30° and a SiNPs content of 3 wt %.

This setup induced a combination of normal and shear forces as the applied force vector decomposed into vertical and horizontal components, depending on the angle. A schematic of the sensor’s internal deformation mechanism under angular loading is illustrated in Figure 5b, where the reversed trapezoidal structure selectively activates different electrodes in contact with the macrodome layer under angular loading. In this design, C1 corresponds to the primary shear direction, C2 to the reverse shear direction, and C3 to the lateral direction relative to shear. Finite element analysis (detailed further in Supplementary Note) was performed on the fabricated sensor subjected to a 20 N force at an angular inclination of 30° (Figure 5c). The results indicated that increasing lateral stress increases asymmetric deformation profiles as the applied shear force to the sensor increased, leading to increasing dissociation of the ionic liquid inside the matrix. This supported our hypothesis that shear forces would result in more distinct directional signal generation, especially when combined with the sensor’s structural anisotropy.

Figure 5d presents the electrical response of sensors containing varying amounts of SiNPs (0, 1, 3, and 5 wt %) under angular compression at 10°, 20°, and 30°, measured at different electrode connections. Here, what we expect is that the applied shear force increases as we increase the angle of the stage from 10° to 30°.

Indeed, all of the sensors showed an electrical response with increasing tendency through the shear force direction to the applied force at the angular stage. The optimal response is characterized by a strong signal at the primary shear direction (C1) and a moderate response at the adjacent side (C3), with a minimal signal at the reverse direction (C2). The sensor containing 3 wt % SiNPs exhibited the highest directional selectivity and linearity, generating signals predominantly at electrode C1 with minimal crosstalk at electrode C2. This difference indicates that mechanical reinforcement at the piezo-ionic interface occurs without overstiffening the film.

However, when the SiNPs content was increased to 5 wt %, the film became excessively rigid, resulting in simultaneous activation of all electrode connections (Figures 5d and S9). This nonselective response under angular loading suggests a loss of directional discrimination, confirming 3 wt % as the optimal filler content for preserving both mechanical responsiveness and directional sensitivity. An example of a directional signal for the sensor with 3 wt % SiNPs at 30° (Figure S10). The force (*F*) applied to fabricated sensors on the angular stage is divided into a shear force component (*F*sinθ) and a normal force component (*F*cosθ) (Figure S11).

Next, Figure 5e shows the plot of the resultant signals at a fixed angle with different electrode connections under increasing forces. The electrode corresponding to the shear direction (C1) exhibited the highest signal amplitude and linear response with increasing force. The side-direction electrode (C3) showed a moderate increase, while the reverse-direction electrode (C2) remained largely unresponsive. These results collectively demonstrate that the designed sensor architecture effectively resolves directional shear input under complex loading conditions.

### Applications for the Smart Shoe Insole System

To demonstrate the practical applicability of the fabricated piezo-ionic shear force sensors, we integrated the sensor into a commercial shoe insole for real-world motion sensing (Figure 6a). The sensor was embedded in the forefoot region, where pressure and shear forces are most pronounced during human gait. Shoe insole integrated with the fabricated sensor is an assembled structure of two different layers, which are the top layer and the bottom layer (Figure 6b). The top layer consists of a reversed trapezoidal porous film connected to five electrodes: four assigned to the directional slope regions (for shear sensing) and one located centrally for detecting normal force. The bottom layer contains a macrodome-patterned porous film with a single ground electrode. Four electrodes are assigned distinct names based on their positions relative to the primary shear direction, which is from the rearfoot toward the forefoot. The bottom-most electrode that responds to forward shear force is named C1 (front). The electrode opposite to C1 that responds to reversed shear force is named C2 (back). The electrodes on each side are named C3 (right) and C3 (left) according to their responding direction. In counterclockwise order, the electrodes are named C2 (back), C3 (left), C1 (front), and C3 (right).

**Figure 6:**
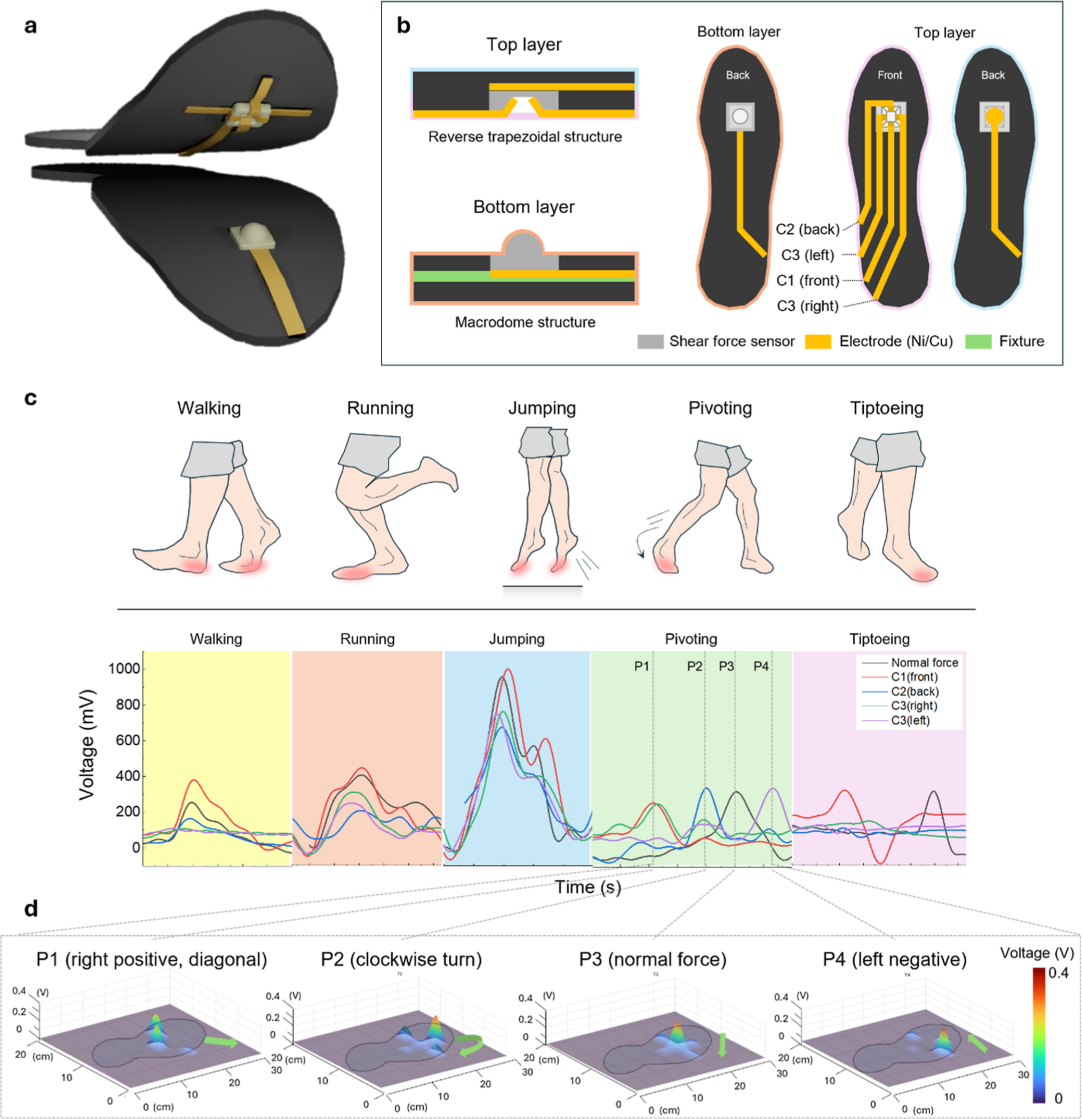
Shoe insole design and deformational behavior. (a) Schematics of the shoe insole with the shear force sensor mounted inside the shoe insole. (b) Schematics showing the electrode connection of our sensor inside the shoe insole. (c) Sensor response to different foot motions (walking, running, jumping, pivoting, and tiptoeing) showing different peak intensities and signals at different connections (C1, C2, C3, and normal force). (d) 3D contour imaging of shear force at pivoting motion from the data acquired by our sensor.

Since the shoe insole used in the application is composed of soft foam, the fixture (green layer in Figure 6b) is attached to the bottom of the macrodome structure, ensuring that the pressure applied to the fabricated sensor is transmitted to the sensor part. The two structured layers were stacked such that the directional and normal electrodes could selectively respond to applied forces through interfacial contact. Once assembled, the system allowed for the simultaneous detection of both normal and shear forces through signal acquisition from specific electrode connections (Figure S12).

To evaluate sensor response under different locomotion types, various foot motions—including walking, running, jumping, pivoting, and tiptoeing—were performed on the insole-integrated system, and corresponding signals were detected (Figure 6c).

Overall, the signal patterns among different connections are similar at the first three motions: walking, running, and jumping. This is because the motion of plantar movement, which induces the normal and shear forces to the forefoot part, is similar in those three different motions. The only difference among those three motions is signal intensities, which are related to applied load and angles. Those differences resulted in greater signal intensity across both normal and shear channels at jumping motion compared with walking motion. Directional response was further validated during pivoting movements. Pivoting motion is applied to the upper right at first, with the diagonal direction of plantar motion, pushing vertically, and then twisted clockwise. Aligning with the deformation by timely different motions, the voltage peaks shifted across connections from C1 (front) and C3 (right) to C2 (back) and C3 (left), demonstrating the sensor’s ability to distinguish changes in shear direction. During tiptoeing, two different signals are dominant at different times of response, which are the initial shear force signals toward the front, followed by normal force signals, confirming the sequential loading detected by the sensor system. The stability of the fabricated sensor for sustained monitoring within a shoe insole is further confirmed by the multiple measurements conducted across various motions (Figure S13). Throughout these repeated trials, stable peaks were consistently generated for each specific motion.

To demonstrate the force distribution during pivoting motion, we divided the movement into four distinct phases: P1 (rightward positive shear with a diagonally positioned plantar surface), P2 (clockwise rotational movement), P3 (vertical pushing force), and P4 (leftward negative shear during deceleration and stopping). Figure 6d presents a 3D contour plot visualizing the magnitude and direction of the shear forces generated by the sensor array during pivoting motion. The spatial mapping clearly distinguishes force distribution, enabling directional shear pressure visualization and assessment of localized plantar dynamics at different time regions. In detail, as the pivoting motion is applied to the shoe insole, different electrodes respond at specific phases of the movement. During the abrupt stopping motion in the right-positive diagonal direction (P1), electrodes C1 (front) and C3 (right) are activated. In the clockwise turning phase (P2), signals are observed from C2 (back) and both C3 (right, left) electrodes. When a vertical pushing force is applied to initiate the stopping motion (P3), only the normal force electrode responds. Finally, during the leftward negative deceleration phase (P4), the C3 (left) electrode is selectively activated.

Overall, the insole-integrated sensor accurately captured dynamic variations in both plantar pressure and shear forces across a range of real-world locomotion scenarios. The ability to distinguish directional shear inputs and temporal force profiles under complex loading conditions demonstrates the versatility of sensing detection.

## Conclusions

In this study, we fabricated a piezo-ionic shear force sensor with a porous, hierarchically structured matrix and demonstrated its integration into a smart shoe insole capable of simultaneously detecting complex normal and shear forces. Leveraging a piezo-ionic mechanism, our TPU/ionic-liquid composite materials generate a signal from ionic-pair dissociation under mechanical deformations. Embedding a porous network within this matrix amplifies both sensitivity and dynamic range by permitting greater, gradual compression of porous morphology composed of nanoparticle–micropore–macrostructures that remain highly deformable and preserve high ion mobility, leading to improved sensitivity and linearity.

The novel design of two-layered architecture, comprising a macrodome base and a reversed trapezoidal upper structure, offers anisotropic mechanical deformation paths, which in turn enable selective activation of electrodes under different directional loads. This geometrically guided deformation, combined with the intrinsic characteristics of the piezo-ionic effect, facilitates precise differentiation of shear direction and magnitude without the need for a power supply and complex signal processing algorithms or multisensor arrays. The addition of SiNPs served a dual purpose. At optimized concentrations (3 wt %), the particles enhanced pore wall rigidity, reducing early signal saturation, while maintaining high film compliance for high-resolution force transduction, as shown in the compression test and shape recovery test.

These shear force sensors demonstrated robust performance under realistic conditions including preload-induced shear, oblique force application, and dynamic foot motion. The angular stage measurements further validated the directional selectivity and linearity of the sensor across a range of incident angles. This sensor demonstrates a sensitivity of 0.229 mV kPa^–1^ over a linear sensing range to 630 kPa, significantly broader than the previously reported sensing range of 300 kPa^17^ (Table S1). Although sensors utilizing triboelectric mechanisms exhibit higher sensitivity,^21^ they saturate at much lower applied pressures, compromising balanced sensitivity and extended operational range. To summarize, Table S2 offers a comparative analysis highlighting our sensor’s comparable sensitivity of 0.567, 0.504, 0.229, and 0.293 mV kPa^–1^ (at 0–35, 35–61, 61–504, and 504–633 kPa regions, respectively) and broad operating range against existing piezo-ionic technologies. Integrated within an insole, the sensor reliably captured variations in plantar shear and normal forces associated with different gait activities such as walking, running, and pivoting, as well as subtle actions like tiptoeing.

Importantly, this sensor operates without an external power supply, relying on the self-generated potential from the mechanical deformation of the piezo-ionic interface. This attribute significantly reduces power requirements and simplifies the system architecture, making it particularly suitable for wearable applications that demand long-term operation and lightweight integration. Taken together, our findings offer a viable route toward replacing bulky gyroscope-based or multiarray normal force insole systems with a compact, single-sensor platform capable of resolving complex plantar pressure distributions relevant to gait analysis, athletic performance monitoring, rehabilitation assessment, and personalized orthotic feedback systems. Future work will focus on scaling this technology for high-resolution sensor arrays, developing wireless data transmission modules, and integrating real-time gait analysis algorithms for clinical and athletic applications. Moreover, the large force range sensor system’s sensitivity and spatial directional resolution open opportunities for use in intelligent wearable robotics, fall prevention in elderly care, and real-time biomechanical diagnostics.

## Methods

### Preparation of TPU/Ionic Liquid/SiNP Composites

#### TPU Material Information

Thermoplastic urethane with a Shore A hardness of 92, a tensile strength of 44.1 MPa, and a tensile stress of 11.1 MPa (at 100% strain) was selected (ELLAS K-490AB, Kolon Industries).

#### Fabrication of Composite Materials from TPU with Ionic Liquid and SiNPs

1-Ethyl-3-methylimidazolium bis­(trifluoromethylsulfonyl)­imide (EMIM-TFSI, Sigma-Aldrich) and fumed SiNPs (average diameter: ∼200–300 nm, Sigma-Aldrich) were used. A solvent mixture was prepared by dissolving 4.0 g of *N*,*N*-dimethylformamide (DMF) with 0.4285 g of EMIM-TFSI and 0.0101 g of SiNPs. The mixture was sonicated to ensure a homogeneous dispersion of the SiNPs. Subsequently, 1.0 g of TPU pellets were added, and the solution was stirred overnight at 70 rpm to obtain a uniform viscous solution.

#### Fabrication of Reverse-Patterned Molds

The surface patterns used to create the macrodome and reverse trapezoidal features were prepared via replica molding. A master structure was 3D-printed by using a fused filament fabrication (FFF) printer (Sketch Large, MakerBot). Before molding, the surface of the 3D-printed mold was treated with oxygen plasma (Harrick Plasma) for 5 min to improve PDMS adhesion. The mold was secured in a Petri dish with double-sided tape. A PDMS mixture (Sylgard 184, Dow Corning; base: curing agent = 10:1) was poured over the mold (20 g total) and degassed for 10 min. The PDMS was then cured on a hot plate at 50 °C until it was fully cross-linked.

#### Fabrication of Porous Patterned Films

To create the porous piezo-ionic film, 1.0 g of the TPU/IL/SiNPs composite solution was poured onto the patterned PDMS mold. The mold was placed under vacuum for 1 h to remove entrapped air and ensure complete penetration into the mold structure. The cast mold was then submerged in a DI water-filled desiccator for 24 h to facilitate solvent exchange from DMF to water, inducing spontaneous phase separation and pore formation. After the solvent exchange, the porous film was gently removed, rinsed in DI water, and dried at ambient conditions for another 24 h.

#### Electrode Attachment

To enable electrical signal acquisition, a liquid metal paste was used to establish connections between the patterned porous film and Ni–Cu foil electrodes. In the reversed trapezoidal film, four electrodes were attached to the angled walls of the slopes for shear force detection and one electrode was positioned at the top center for normal pressure sensing. The macrodome-structured bottom layer was equipped with a single ground electrode.

### Mechanical and Electrical Characterization

Tensile/compression tests were performed using a universal test machine (UTM, EZ-SX, Shimadzu) with a 500 N load cell. The TPU/IL/SiNPs composite solutions were cast into a dog-bone-shaped negative PDMS mold, followed by solvent exchange and drying, the same procedure as that used to prepare the patterned film. The prepared dog-bone-shaped specimens had dimensions of 3 mm in width and 35 mm in gauge length. The thickness of specimens was the average thickness from three different locations. The strain rate was 10 mm/min. For the compression test, square specimens with 12.5 mm × 12.5 mm (width × length) dimensions were cut from the film. The specimens were compressed to 80% of their original thickness at a rate of 3 mm/min to measure compressive properties. Compression measurements began after applying a 0.01 N preload with a strain rate of 1 mm/min.

Normal force measurements were performed using a MARK-10 testing machine (model ESM303). The testing system comprised a 0.8 cm-radius round indenter made from a hard polyurethane-based resin (Smooth-Cast 300), which applied controlled loading and unloading cycles to the sensor. The sensor was fixed onto a stage, ensuring that both the reverse trapezoidal and macrodome layers remained in consistent contact throughout the test. The loading–unloading speed was fixed at 250 mm/s. The sensor was mounted on a rigid stage with both patterned layers aligned and secured.

Shear force under preload was evaluated using a horizontal motion setup consisting of a syringe pump (flow rate equivalent to 1.74 mm/s linear speed) and preload masses (100 g, 500 g, 1 kg). The sensor was fixed on the base, while the preload and horizontal motion were applied from the syringe pump stage.

Angular force measurements were conducted on custom-fabricated inclined platforms (10°, 20°, 30°) created by 3D printing using the same filament-based method. The sensors were mounted on each inclined surface and compressed using the MARK-10 system to simulate combined shear and normal loading.

Finite Element Method (FEM) simulations of deformation under various loading conditions were carried out via ANSYS Mechanical 2024 R2 using the static structural module to visualize internal strain and validate contact mechanics. The mesh size was set to 0.5 mm, with adaptive sizing on the edges to improve the conformal frictional contact between the elements. The boundary conditions were established as a fixed support on the bottom face of the macrodome piece and a 20 N vertical load on the top surface of the reverse trapezoidal element, varying its angle to 30° for the shear force simulations.

### Contour Mapping of Normal/Shear Force at Plantar by MATLAB

Contour mapping was conducted using MATLAB (MathWorks, USA) to process and visualize peak distribution data. Peak values (right, up, down, left) and normal position were used to visualize 3D surface representations with the “surf” command in MATLAB. We used Modified Akima cubic Hermite (“makima”) interpolation to generate smooth surface visualizations without overshoots of each data value, thereby avoiding compromise to data integrity.

## Supplementary Material



## Notes

Digital picture of porous film; loading–unloading cycle comparison; sensor response at lower pressure region; contact area of round indenter; plot of normal measurement result; response of different electrodes at normal force; response of different electrodes at increasing shear force; response of different electrodes at shear force; loading–unloading cycle of different sensors at angular stage; response of the fabricated sensor at fixed angle; schematic of angular force; picture of the fabricated shoe insole; and graphs of repeated motion monitored (PDF)
