# Supplementary material for: Piezo-ionic Materials and Structures for Complex Shear Field Monitoring
Source: ACS Appl Mater Interfaces. 2025 Jul 7;17(28):41350–60. doi: 10.1021/acsami.5c10061 (PMC12278252; doi:10.1021/acsami.5c10061)
Supplement: Supplementary file 1 [file am5c10061_si_001.pdf]

Supporting Information

Piezo-ionic Materials and Structures for Complex Shear Field Monitoring

*Dong-hee Kang†, Jinyoung Kim†, Sergio Gonzalez Munoz, Jisoo Jeon, Sehyun Park, and Vladimir V. Tsukruk**

School of Materials Science and Engineering, Georgia Institute of Technology, Atlanta, GA, 30332, USA

*Corresponding Author

E-mail: Vladimir V. Tsukruk vladimir@mse.gatech.edu

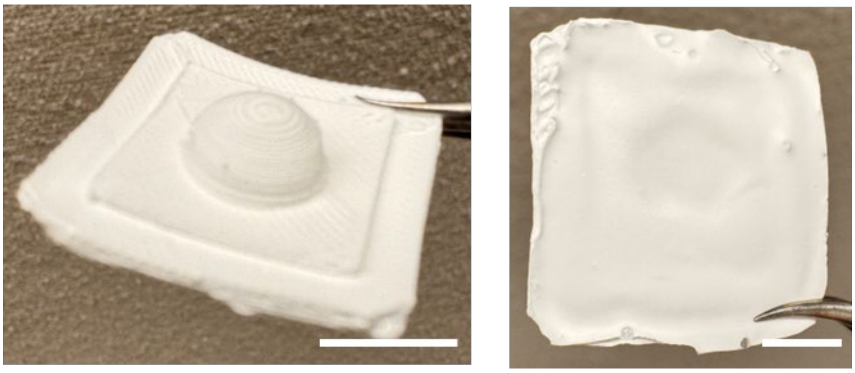

Figure S1. Fabricated porous film having macrodome structure – left (top) and right (bottom) (scale bar = 1 cm)

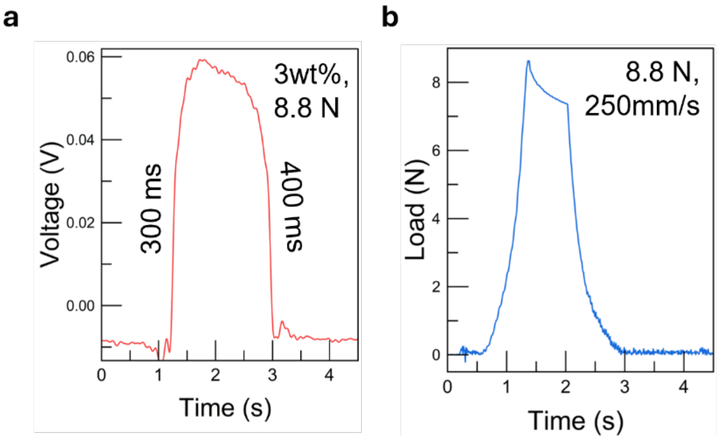

Figure S2. Response to a single loading-unloading cycle of (a) fabricated sensor with 3 wt% of SiNPs and (b) commercial force sensor.

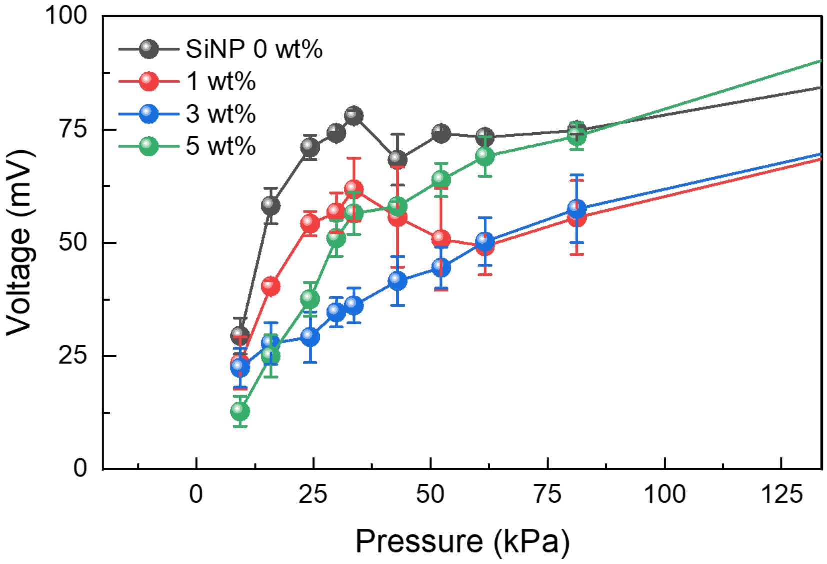

Figure S3. Voltage output result of the fabricated sensor system with different amounts of SiNPs. (a) Result of pressure at lower range (<100 kPa)

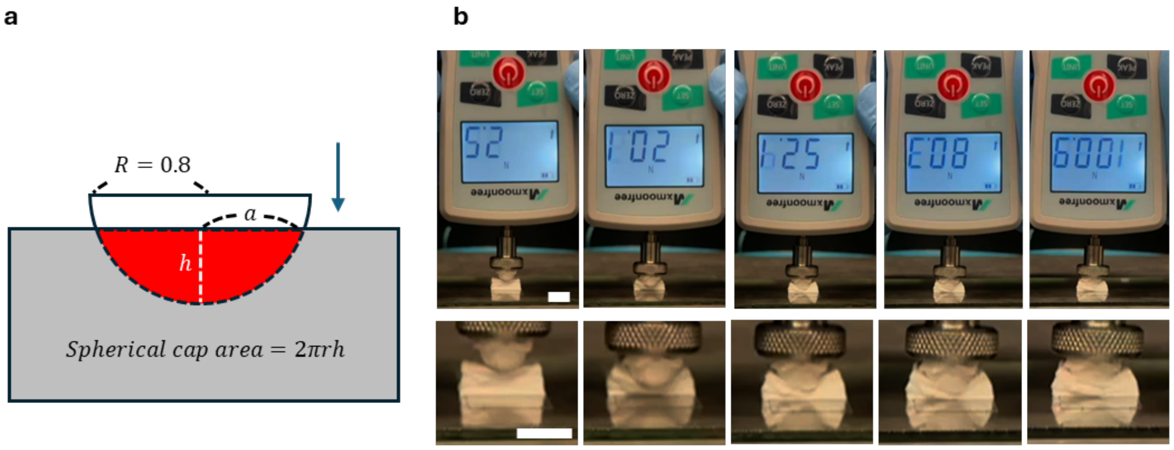

c

Force (N)	Cap distance (a) (cm)	Area (cm^2)	Pressure (kPa)
2.5	0.23	1.07	23.3
20.1	0.30	1.25	230.3
52.1	0.34	1.38	449.2
80.3	0.40	1.59	504.2
100.9	0.40	1.59	633.5

Figure S4. Contact area calculation with the round indenter. (a) Schematic of contact area of round indenter (b) Picture of compressing porous film at different force (scale bar = 1 cm). (c) Applied pressure is calculated based on the contact area of round shape

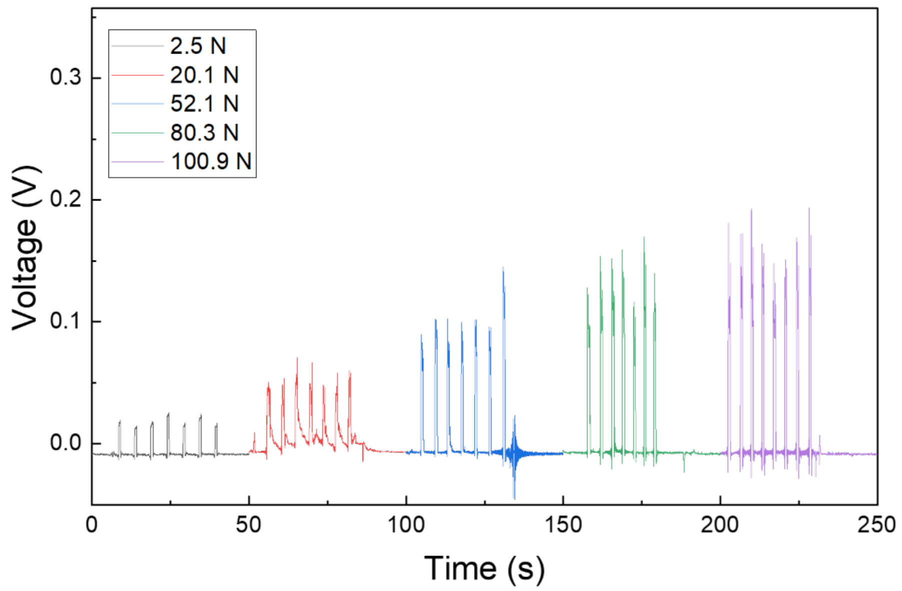

Figure S5. Normal force measurement results at differently applied force with sensors containing 3 wt% of SiNPs.

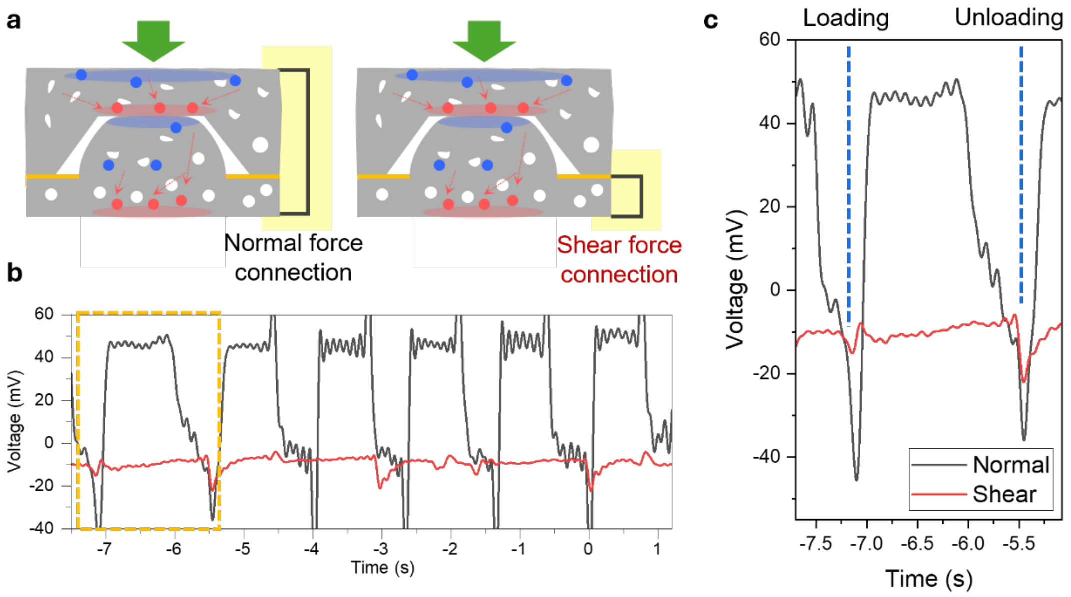

Figure S6. Response at different electrode connection at applying normal force. (a) Schematics indicating two different electrode connections at applying normal force. (b) Plot of signals

generated in normal and shear force connection electrode connections (c) Magnified view of orange box in (b), at loading-unloading cycle, the unwanted contact of shear force connection and macrodome does not happen at normal force measurement.

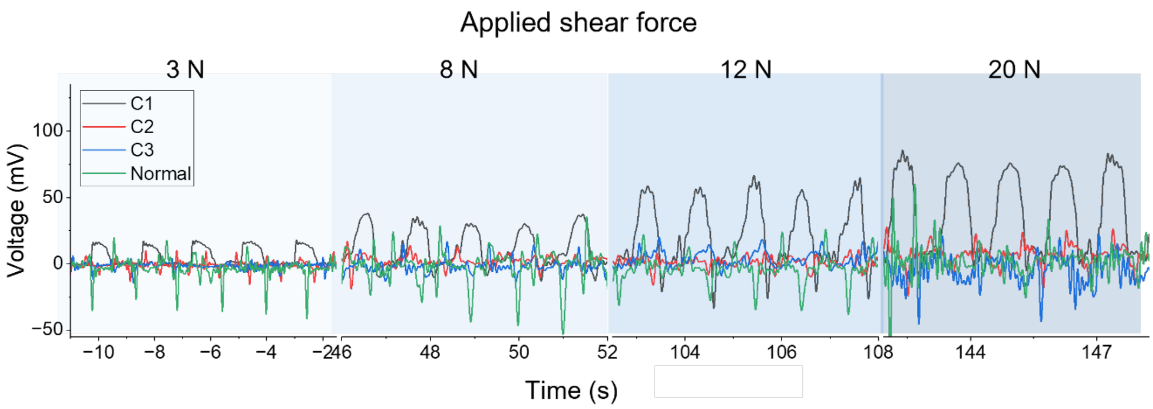

Figure S7. Plot of output voltage at different electrode connections at applying increasing shear force from 3, 8, 12, 20 N, applied preload condition is 0.98 N.

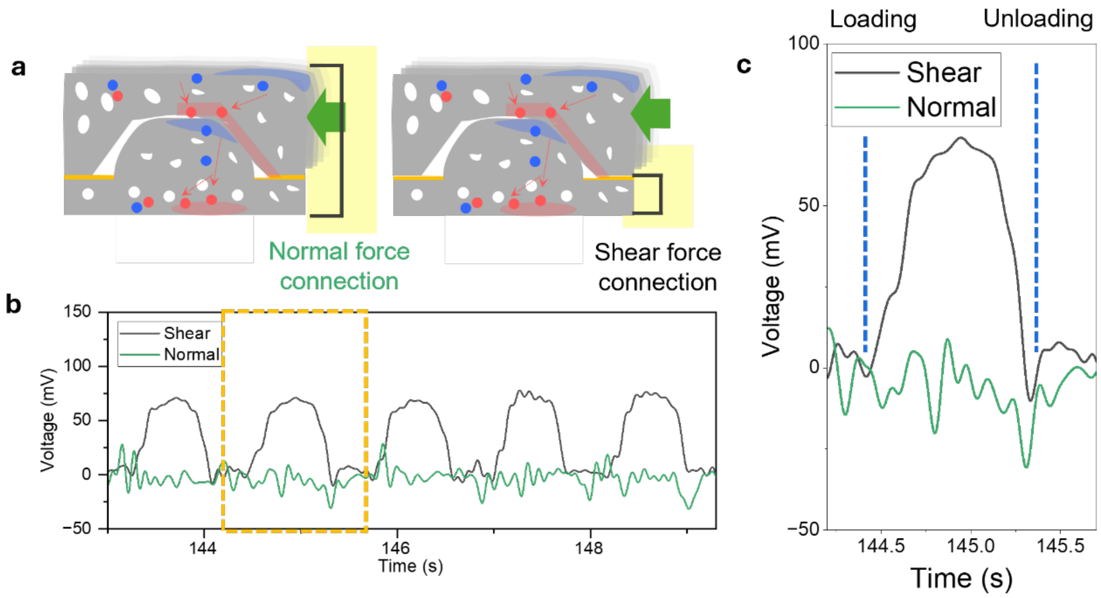

Figure S8. Response at different electrode connection at applying shear force. (a) Schematics indicating two different electrode connections at applying shear force. (b) Plot of signals generated in normal and shear force connection electrode connections (c) Magnified view of orange box in (b), at loading-unloading cycle, the unwanted contact of normal force

connection and macrodome does not happen at shear force measurement.

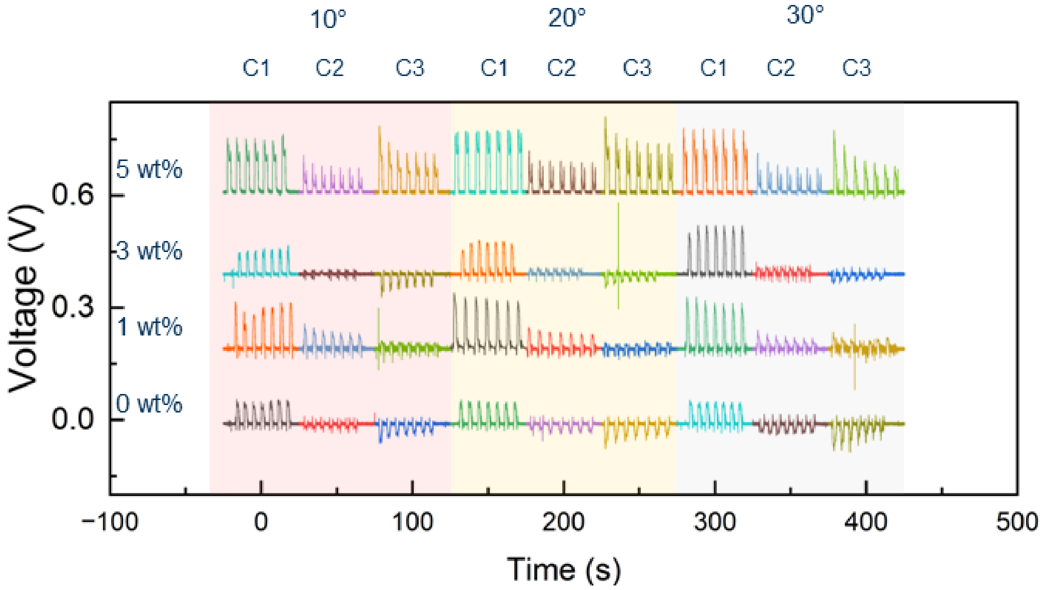

Figure S9. Plot of loading-unloading response at different electrode connections (C1, C2 and C3) of fabricated shear force sensors with different amounts of SiNPs content (0, 1, 3, 5 wt%) at increasing angles of angular stage to 10, 20, and 30°.

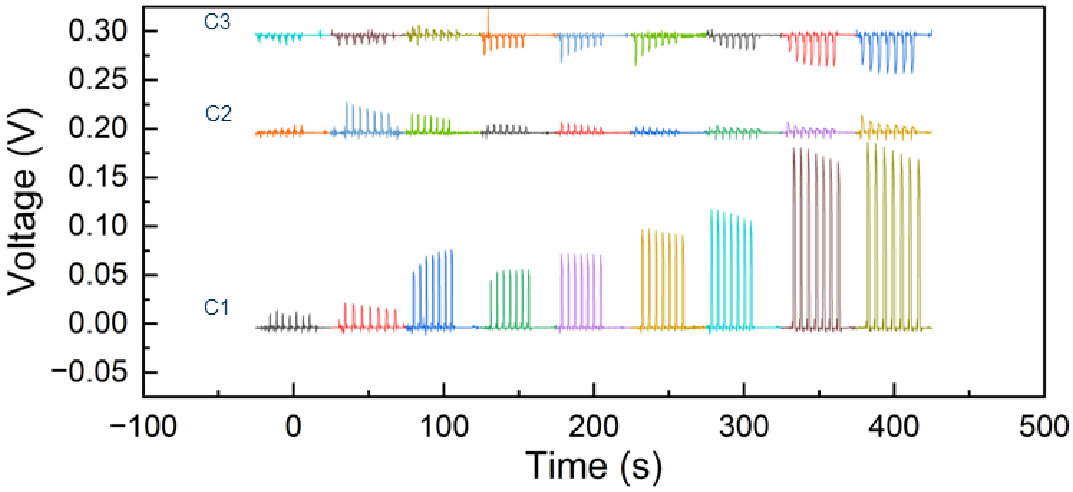

Figure S10. Directional response of fabricated shear force sensor at a fixed angle of 30° and SiNPs content of 3 wt%.

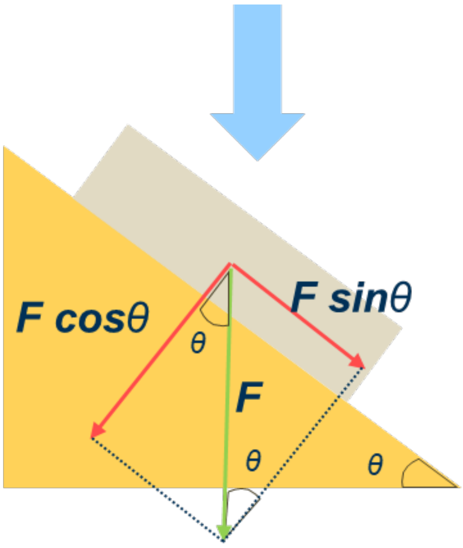

Figure S11. Schematics of angular force divided into shear force ($Fsin\theta$) and normal force ($Fcos\theta$)

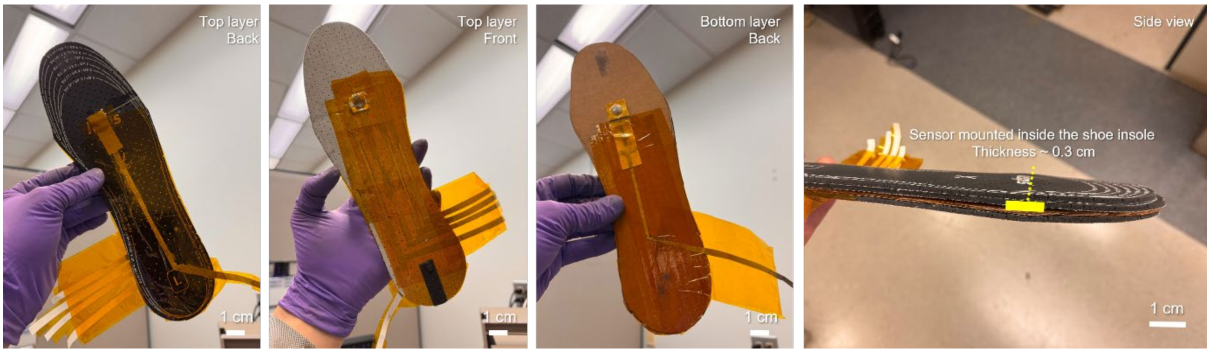

Figure S12. Picture of fabricated shoe insole integrated with shear force sensor. Pictures in order of top layer (back), top layer (front), bottom layer (back), and side view of shoe insole.

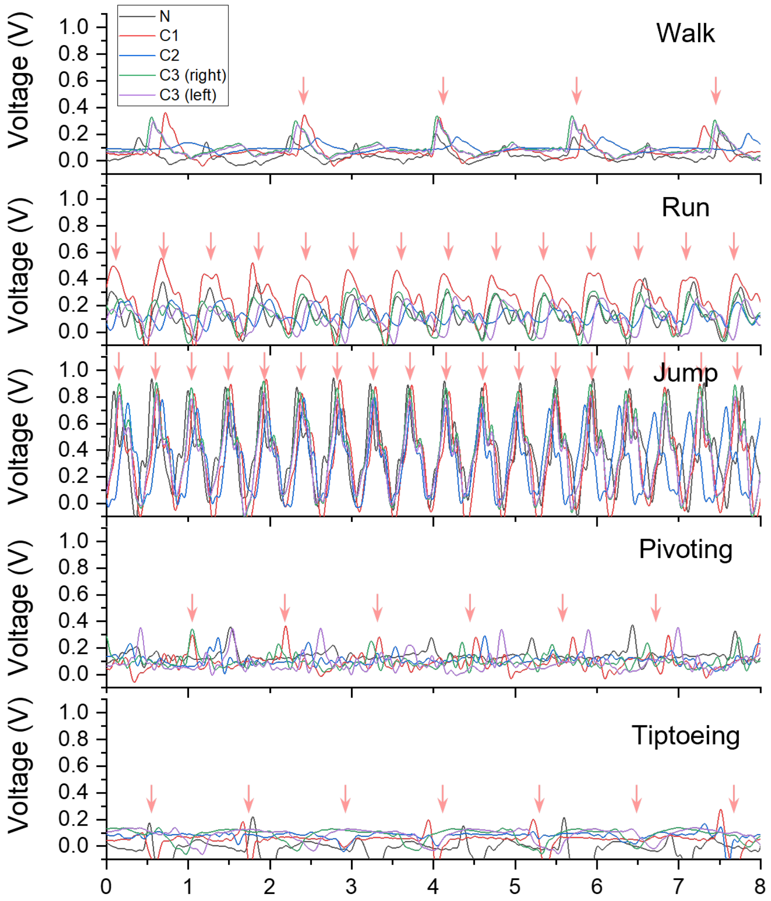

Figure S13. Graphs showing repeated measurements at different foot postures. The red arrows indicate the start point of each motion.

Supplementary Note. Finite Element Method (FEM)–based simulation of fabricated sensor's deformation

The mechanical response of the interlocked structure was simulated via ANSYS Mechanical 2024 R2 finite element modelling (FEM) using the static structural module. The intrinsic Young's moduli and Poisson ratios of the structures were chosen from literature[1] using Halpin-Tsai reinforcement model, as E_{TPU} = 2 MPa v_{TPU} = 0.2 and E_{tape} = 2.76 GPa and v_{tape} = 0.34 and the frictional contacts were attributed to accurately represent the experimental behavior. For normal force simulations, an indenter was added to limit the vertical pressure to a smaller area on the top surface of the trapezoidal structure, while the shear force was applied with a directional vector at of the same top electrode surface. By applying a constant force of 20 N, we obtained the real-time monitoring of the local deformation and stress as well as their distribution over the structure geometry at both normal and shear conditions.

FEM based simulation of deformation at applying normal force

In Figure 3d, under pure normal indentation of 20 N, the deformation map expectedly shows the largest displacements (1.44 mm) of the porous structures on the top surface of the reversed trapezoidal structure, where the force is applied. Nevertheless, this deformation is transmitted almost equally along the thin separation of porous TPU to its contact region with the macrodome. Thickness reduction is observed on both the thin horizontal wall of the top electrode and the macrodome due to the high normal compression, which translates into an increased symmetrically centered interfacial area of ≈ 28.27 mm^2 and a stress of 3.83 MPa along the indentation axis. In turn, this causes a greater density of ionic distribution. Deformation is transmitted towards the edges where the flat wall transitions into the sloped trapezoidal sides, experiencing concentrated bending and forming flexion points. Such strain results from a vertical compression translated into gradually localized downward flexion, rather than uniform compression of the entire sloped region. Thus, under vertical loading, the sensor's architecture shows preferential bending of the trapezoid top edge and expansion of the central contact zone rather than a full engagement of the trapezoidal slopes, limiting shear activation. This design ensures that pure compression predominantly activates the central electrode, minimizing crosstalk with shear-sensitive contacts.

FEM based simulation of deformation at applying angular force

Figure 5c captures the sensor response to 20 N angular loading at 30º with respect to the vertical axis, introducing both normal and lateral stress components. The simulation shows asymmetric deformation with lateral displacement along the shear axis. A maximum displacement of 2.83 mm is obtained at the top left edge of the trapezoidal part; however, the left sloped internal face has a bigger contribution to the deformation compared to the pure normal force condition, due to its shear force contact with the side of the macrodome. Also, given the higher normal component of the angular force, the internal flat surface of the trapezoid also causes a lateral deformation with a higher magnitude at the top of the macrodome, but, contrary to Figure 3e, this is not symmetrically centered, thus, reorienting the contact mechanics toward one side of the sensor, selectively engaging a subset of the electrodes (C1 and C3). This spatial redistribution directly correlates with the directional signal output observed experimentally, as only the electrodes aligned with the lateral deformation direction generate a strong voltage response, while the reverse-side electrode (C2) remains relatively inactive. This confirms that the FEM simulation captures not only the mechanical behavior but also predicts the electro-mechanical sensing dynamics of the device.

Furthermore, the contact lateral displacement reaches a magnitude on the order of ≈ 0.7–0.9 mm, sufficient to induce differential contact patterns without causing delamination or plastic failure. The gradient of stress across the sloped surface implies progressive engagement of the slanted interface, further supporting the interpretation that shear stimuli translate into contact asymmetry rather than simple horizontal translation. This is key to avoiding crosstalk between shear and normal signals, as the deformation pathways diverge depending on the loading direction.

Table S1. Performance comparison with previously reported shear force sensors

Reference	Design	Mode	Range	Sensitivity	Directi on	Durability (cycles)	Response time	Ref #
Sci. Adv. 2024	3d printed mesh	Capacitive	400 kPa	0.002 kPa^{-1}	4	800	1.1 s	[2]
Adv. Mater. Technol. 2019	Interlocked pyramid	Capacitive, Piezoresistive	50 kPa	0.7 kPa^{-1} (C), 1.93 kPa^{-1}(R)	-	-	69 ms	[3]
ACS Appl. Mater. Interfaces 2023	Dome / planar electrode	Piezoionic	200 kPa	0.0689 nF kPa^{-1}	4	4,000	-	[4]
ACS Appl. Mater. Interfaces 2022	Cone structure	Triboelectric	16 N (40 kPa)	2.97 mV kPa^{-1}	4	10,000	260 ms	[5]
Nat. Commun. 2022	Shielded liquid metal	Capacitive	20 N	2.77 mN fF^{-1} (N), 0.23mN fF^{-1} (S)	2	-	-	[6]
Sci. Adv. 2020	Thin-film transistor array	Piezoelectric	250 mN (N)<br>20 mN (S)	9.93pmV^{-1}	4	-	10 ms	[7]
Sensors and Actuators A 2021	Interlocked dome	Piezoresistive	12 kPa	6.33 kPa^{-1}	3	1,500	59 ms	[8]
**This work**	**Macrodome / reverse trapezoid**	**Piezoionic**	**633.5 kPa**	**0.567 mV kPa^{-1} (~ 35 kPa)**<br>**0.504 mV kPa^{-1} (35-61 kPa)**<br>**0.229 mV kPa^{-1} (61-504 kPa)**<br>**0.293 mV kPa^{-1} (504-633 kPa)**	**4**	**5,000**	**300 ms**	

*C: Capacitive, R: Resistive, N: Normal force, S: Shear force

Table S2. Sensitivity of recently reported piezo-ionic sensors

Reference	Materials	Ionic component	Sensitivity	Pressure range	Ref #
Chemical Engineering Journal, 2024	Stacked filter paper	LiCl	0.73 kPa^{-1} (0.1–4 kPa)<br>0.058 kPa^{-1} (4–50 kPa)<br>0.023 kPa^{-1} (50–100 kPa)	100 kPa	[9]
Smart Mol. 2024	Polyurethane-urea	EMIM-Cl	7.03 kPa^{-1} (0–15 kPa)<br>2.38 kPa^{-1} (15–55 kPa)<br>0.95 kPa^{-1} (55–90 kPa)	90 kPa	[10]
Science, 2022	Poly acrylic acid – acryl amide	Poly acrylic acid – acryl amide	0.11 mV kPa^{-1}	20 kPa	[11]
Electroactive Polymer Actuators and Devices (EAPAD), San Diego, California, April 2015. 2016	Polyurethane hydrogel	Nafion	0.3 mV kPa^{-1}	30 kPa	[12]
Adv. Funct. Mater. 2023	hydrogel	SnSe nanosheets	1.78 mV kPa^{-1}	3 N	[13]
Adv. Mater. 2024	PVDF-HFP	EMIM-TFSI	6.0 mV kPa^{-1}	15 kPa	[14]
**This work**	Porous thermoplastic urethane / SiNPs	EMIM-TFSI	0.567 mV kPa^{-1} (~ 35 kPa)<br>0.504 mV kPa^{-1} (35-61 kPa)<br>0.229 mV kPa^{-1} (61-504 kPa)<br>0.293 mV kPa^{-1} (504-633 kPa)	633 kPa	

References

[1] Halpin, J. C. The Halpin-Tsai Equations: A Review. *Polym. Eng. Sci.* **1976**, *16*, 5

[2] Berman, A.; Hsiao, K.; Root, S. E.; Choi, H.; Ilyn, D.; Xu, C.; Stein, E.; Cutkosky, M.; DeSimone, J. M.; Bao, Z.; Additively manufactured micro-lattice dielectrics for multiaxial capacitive sensors. *Sci. Adv.* **2024**, 10, eadq8866

[3] Choi, D.; Jang, S.; Kim, J. S.; Kim, H.-J.; Kim, D. H.; Kwon, J.-Y. A Highly Sensitive Tactile Sensor Using a Pyramid-Plug Structure for Detecting Pressure, Shear Force, and Torsion. *Adv. Mater. Technol.* **2019**, 4, 1800284

[4] Suh, W.; Ki, K.; Kim, T.; Choi, H.; Lee, A.; Jeong, U. Shear-Pressure Decoupling and Accurate Perception of Shear Direction in Ionic Sensors by Analyzing the Frequency-Dependent Ionic Behavior. *ACS Appl. Mater. Interfaces* **2023**, 15, 51538-51548

[5] Zhang, W.; Xi, Y.; Wang, E.; Qu, X.; Yang, Y.; Fan, Y.; Shi, B.; Li, Z. Self-Powered Force Sensors For Multidimensional Tactile Sensing. *ACS Appl. Mater. Interfaces* **2022**, 14, 17, 20122–20131

[6] Aksoy, B.; Hao, Y.; Grasso, G.; Digumarti, K. M.; Cacucciolo, V.; Shea, H. Shielded soft force sensors. *Nat. Commun.* 2022, *13*, 4649

[7] Oh, H.; Yi, G.-C.; Yip, M.; Dayeh, S. A. Scalable tactile sensor arrays on flexible substrates with high spatiotemporal resolution enabling slip and grip for closed-loop robotics. *Sci. Adv.* **2020***,* eabd7795

[8] Chen, S.; Bai, C.; Zhang, C.; Geng, D.; Liu, R.; Xie, Y.; Zhou, W. Flexible piezoresistive three-dimensional force sensor based on interlocked structures. *Sensors and Actuators A* **2021**, 330, 112857

[9] Huang, Q.; Jiang, Y.; Duan, Z.; Wu, Y.; Yuan, Z.; Zhang, M.; Tai, H. Ion gradient induced self-powered flexible pressure sensor. *Chem. Eng. J.* **2024**, *490*, 151660.

[10] Wang, X.; Liu, T.; Sun, F.; Zhang, J.; Yao, B.; Xu, J.; Fu, J. Highly tough, crack-resistant and self-healable piezo-ionic skin enabled by dynamic hard domains with mechanosensitive ionic channel. *Smart Mol.* **2024**, *2*, e20240008.

[11] Dobashi, Y.; Yao, D.; Petel, Y.; Nguyen, T. N.; Sarwar, M. S.; Thabet, Y.; Ng, C. L. W.; Glitz, E. S.; Nguyen, G. T. M.; Plesse, C.; Vidal, F.; Michal, C. A.; Madden, J. D. W. Piezoionic mechanoreceptors: Force-induced current generation in hydrogels. *Science* **2022**, *376*, 502.

[12] Dobashi, G. A. Y.; Sarwar, M. S.; Cretu, E.; Madden, J. D. W. Transparent and conformal 'piezoionic' touch sensor. Presented at *Electroactive Polymer Actuators and Devices (EAPAD)*, San Diego, California, April 2015. *Proc. SPIE* **2016**, *9798*, 979806.

[13] Li, F.; Cai, X.; Liu, G.; Xu, H.; Chen, W. Piezoionic SnSe nanosheets–double network hydrogel for self-powered strain sensing and energy harvesting. *Adv. Funct. Mater.* **2023**, *33*, 2300701.

[14] Zhu, W.; Wu, B.; Lei, Z.; Wu, P. Piezoionic elastomers by phase and interface engineering for high-performance energy-harvesting ionotronics. *Adv. Mater.* **2024**, *36*, 2313127.
